# The long noncoding RNA *LINC15957* regulates anthocyanin accumulation in radish

**DOI:** 10.3389/fpls.2023.1139143

**Published:** 2023-02-27

**Authors:** Huping Tan, Xiaobo Luo, Jinbiao Lu, Linjun Wu, Yadong Li, Yueyue Jin, Xiao Peng, Xiuhong Xu, Jingwei Li, Wanping Zhang

**Affiliations:** ^1^ College of Agriculture, Guizhou University, Guiyang, China; ^2^ Institute of Vegetable Industry Technology Research, Guizhou University, Guiyang, China; ^3^ Guizhou Institute of Biotechnology, Guizhou Province Academy of Agricultural Sciences, Guiyang, China

**Keywords:** radish, anthocyanin, lncRNA, transcriptome, differentially expressed genes (DEGs)

## Abstract

Radish (*Raphanus sativus* L.) is an important root vegetable crop belonging to the Brassicaceae family. Anthocyanin rich radish varieties are popular among consumers because of their bright color and high nutritional value. However, the underlying molecular mechanism responsible for skin and flesh induce anthocyanin biosynthesis in transient overexpression, gene silencing and transcriptome sequencing were used to verify its function in radish anthocyanin accumulation, radish remains unclear. Here, we identified a long noncoding RNA *LINC15957*, overexpression of *LINC15957* was significantly increased anthocyanin accumulation in radish leaves, and the expression levels of structural genes related to anthocyanin biosynthesis were also significantly increased. Anthocyanin accumulation and expression levels of anthocyanin biosynthesis genes were significantly reduced in silenced *LINC15957* flesh when compared with control. By the transcriptome sequencing of the overexpressed *LINC15957* plants and the control, 5,772 differentially expressed genes were identified. A total of 3,849 differentially expressed transcription factors were identified, of which MYB, bHLH, WD40, bZIP, ERF, WRKY and MATE were detected and differentially expressed in the overexpressed *LINC15957* plants. KEGG enrichment analysis revealed the genes were significant enriched in tyrosine, L-Phenylalanine, tryptophan, phenylpropanol, and flavonoid biosynthesis. RT-qPCR analysis showed that 8 differentially expressed genes (DEGs) were differentially expressed in *LINC15957*-overexpressed plants. These results suggested that *LINC15957* involved in regulate anthocyanin accumulation and provide abundant data to investigate the genes regulate anthocyanin biosynthesis in radish.

## 1 Introduction

Color is one of the most significant quality traits of plants, and is the most adaptive phenotypic trait in the evolution of plants. Anthocyanin are an important pigment that gives plants their color, and color trait is one of the important research directions of plant genetic improvement (Dai and Hong, 2016). Anthocyanin as common antioxidant substances plays important role in scavenging free radicals, accelerating human metabolism, protect vision, smooth blood sugar, fight obesity and inflammation, and delaying aging and preventing cardiovascular disease (Nomi et al., 2019). In addition, anthocyanin is involved in many biological processes, such as plant environmental stress response, pathogenic bacteria and insect stress response. Anthocyanin synthesis is regulated by several transcription factors, of which a protein complex (MBW) consisting of R2R3-MYB, bHLH and WD40 transcription factors binds to the promoters of structural genes in plants (Ramsay and Glover, 2005).

Radish (*Raphanus sativus* L., 2n = 2x = 18) is an important vegetable crop. Radish has plentiful germplasm resources and a long history of cultivation. After long-term exposure to natural and artificial selection, different varieties with different skin and flesh colors have been formulated. Among them, anthocyanin-rich radish varieties are popular among consumers because of their bright color and high nutritional value. Anthocyanin have been widely used as natural pigments because of their good thermal stability and beneficial antioxidant activity in radish (Rahman et al., 2006; Matsufuji et al., 2007). *AtPAP1/2* is a key transcription factor regulating anthocyanin biosynthesis in Arabidopsis. The homologous gene of *AtPAP1/2* in radish is generally involved in the regulation of anthocyanin biosynthesis in radish, and *RsMYB1* was demonstrated to be a key transcription factor regulating anthocyanin synthesis by heterologous overexpression in Arabidopsis, tobacco, and Petunia (Lim et al., 2016; Ai et al., 2017). A key gene controlling anthocyanin biosynthesis in the fleshy root of radish was mapped by QTL-seq and RNA-seq techniques and validated the function of *RsMYB1* (Wang et al., 2020a). Previous studies found that the homologs of *AtPAP1/2*, *RsMYB41*, *RsMYB117*, and *RsMYB132*, were found in the red radish genome (Muleke et al., 2021). *RsMYB132* were highly expressed in the root bark of red-skinned radish fleshy roots, whereas *RsMYB65* and *RsMYB159* were highly expressed in the root bark of purple-skinned radish fleshy roots, indicating these genes are involved in regulating anthocyanin synthesis in radish fleshy roots. Several key members of the v-myb avian myeloblastosis viral oncogene homolog (MYB), basic helix-loophelix (bHLH) and WRKY families are major drivers of transcriptional changes between purple and green radish (Zhuang et al., 2019). Previous studies showed that *RsGSTF12-1* and *RsGSTF12-2* may participated in anthocyanin transport in carmine radish (Gao et al., 2020a). The expression pattern of *RsTT19* was consistent with key genes of the anthocyanin synthesis pathway in radish (Liu et al., 2019b). Radish multidrug and toxic compound extrusion (MATE) gene family members *RsMATE2*, *RsMATE3*, *RsMATE7*, *RsMATE8*, and *RsMATE9* was participated in radish anthocyanin translocation through phylogenetic tree and expression analysis (M’mbone et al., 2018). However, the molecular mechanism underlying radish anthocyanin biosynthesis in radish remains unclear.

Long noncoding RNAs (lncRNAs) are a class of RNA molecules that do not encode proteins and can interact with proteins, DNA and RNA (Ponting et al., 2009). Eukaryotic genomes encode thousands of lncRNAs, which play important roles in vital biological processes (Wu et al., 2020a). To date, at least four different lncRNA-mediated regulatory mechanisms have been revealed, including target mimicry, transcriptional interference, PRC2-associated histone methylation, and DNA methylation. Although lncRNAs have roles in both the nucleus and cytoplasm, they are mostly found in the nucleus (Chekanova, 2015). LncRNAs have been shown to act as transcriptional regulators and competitive endogenous RNAs (ceRNAs) as molecular cargoes for protein relocalization and as modular scaffolds to recruit the assembly of multiple protein complexes for chromatin modification. Many studies have indicated that lncRNAs have involved in regulating flowering, male sterility, nutrient metabolism, and biotic and abiotic stress responses in plants (Zhang et al., 2013; Liu et al., 2015; Wang et al., 2018). Previous studies found that an endogenous rice lncRNA, *TWISTED LEAF* (*TL*) play a cis-regulatory role on *OsMYB60* in leaf morphological development (Liu et al., 2018a). Silencing of two novel intergenic lncRNAs in tomato, *lncRNA1459* and *lncRNA1840*, resulted in a significant delay in wild-type fruit ripening, suggesting lncRNAs may be important regulators of tomato fruit ripening (Zhu et al., 2015). The apple lncRNA *MSTRG.85814* positively promoted *SAUR32* expression, which then activated proton extrusion involved in the Fe-deficiency response (Sun et al., 2020). In strawberry, 50,601 putative lncRNAs associated with anthocyanin were identified, 68 lncRNAs were differentially expressed and co-expressed with anthocyanin-related mRNAs (Lin et al., 2018). *LNC1* and *LNC2* were identified as targets of *miR156a* and *miR828a* to reduce *SPL9* expression and induce *MYB114* expression, respectively, which lead to increased and decreased anthocyanin content (Zhang et al., 2018). Through Weighted correlation network analysis (WGCNA analysis) miRNA-lncRNA-mRNA expression regulation network construction, and gene function verification, confirmed that lncRNA *MLNC3.2* and *MLNC4.6* are potential targets of *miRNA156a*, and prevented the degradation of *SPL2-like* and *SPL33* by *miR156a* under light induction, promoting the expression of *SPL2*-like and *SPL33* and the accumulation of anthocyanin (Yang et al., 2019). A total of 2,070 co-expressed lncRNA-mRNA pairs were generated, *MdLNC610*, a positive regulator promoting *MdACO1* expression and ethylene biosynthesis, was involved in the regulation of strong light-induced anthocyanin production in apple (Yu et al., 2022). Most of the flavonoid synthesis pathway genes may also be regulated by lncRNAs, and constructed *sly-miR5303*, *stu-miR5303g*, *stu-miR7997a*, and *stu-miR7997c* three “lncRNA-miRNA-mRNA” regulatory networks, including 28 differentially expressed mRNAs and 6 differentially expressed lncRNAs (Zhou et al., 2022). *MdLNC499* in apple pericarp positively regulates the expression of *MdERF109* to promote light-induced anthocyanin biosynthesis (Ma et al., 2021). A specific lncRNA-miRNA-mRNA network was formed in different tissues of apple, and *MSTRG.60895.2*-*mdm*-*miR393*-*MD17G1009000* may be involved in anthocyanin metabolism in fruit (Wang et al., 2022).

Based on our previous lncRNA sequencing results with a high and low anthocyanin content varieties, a long noncoding RNA *LINC15957* was found to be differentially expressed, and selected for further explored. In this study, *LINC15957* was conducted to functional assays by transient overexpression and virus-induced gene silencing technology. The expression of anthocyanin structural genes was validated in *LINC15957* overexpressed plants and control by qRT-PCR. Transcriptome sequencing of *LINC15957* overexpressed plants and control were performed to investigate the differentially expressed genes in the accumulation of anthocyanin. These results provide a theoretical basis for further investigation of the molecular mechanism of lncRNAs involvement in anthocyanin accumulation in radish.

## 2 Materials and methods

### 2.1 Materials and treatments

The full seeds of the high generation inbred plant with red-skin and flesh radish (YZH) were selected and sown in cavity trays (5 × 10). The seedlings were raised in an artificial climate chamber (day temperature 25°C, 16 h; night temperature 16°C, 8 h; humidity 75%), and three true leaves were transplanted into plastic pots (outer diameter 29.2 cm, height 23.5 cm), with 15 pots of each material. The fleshy roots were allowed to expand to about 5 cm. Three plants of test materials were taken (three biological replicates), and two portions of root bark were taken from each plant, 1 g each, wrapped in tin foil and immediately snap-frozen in liquid nitrogen and stored at -80°C. Healthy two-leafed plants were selected as Agrobacterium-mediated transient transformation material for radish.

### 2.2 Total RNA extraction and cDNA synthesis

RNA was extracted from 0.1 g radish root skin using RNAiso Plus (TaKaRa, Beijing, China) liquid nitrogen extraction method. The quality was identified by 1.0% agarose gel electrophoresis, and the RNA concentration and purity were detected by ultra-micro spectrophotometer. cDNA was synthesized using StarScript II RT Mix with gDNA Remover (GeneStar, Beijing, China) according to the reagent instructions. The cDNA was stored for further gene cloning and quantitative real-time PCR (RT-qPCR) analysis.

### 2.3 *LINC15957* overexpression vector construction and plant transformation

The full sequence of *LINC15957* was cloned into the pGreenII 62-SK vectors with restriction endonuclease BamH I and Kpn I. The recombinant plasmids were transformed into Agrobacterium tumefaciens GV3101 by freeze-thaw method. “Degaohongmeizan” radish cotyledons and true leaves were treated by injection according to the method of Fan et al. (2020). The cotyledons injected with pGreenII 62-SK were used as controls. The infiltrated cotyledons were cultured in the dark for 2 days and then transferred to normal light conditions. The coloration of cotyledons near the injection site was closely monitored and photographed. The infiltrated cotyledons with red color were sampled and preserved for anthocyanin content assay and RT-qPCR analysis.

### 2.4 Virus-induced gene silencing

To functionally characterize *LINC15957*, a TYMV-based virus-induced gene silencing (VIGS) system was used (Pflieger et al., 2008; Yu et al., 2018). A palindromic DNA fragment of 80 nt corresponding to *LINC15957* genes was designed and synthesized, and 15-nt homologous sequences corresponding to the vector were introduced to both 3′ and 5′ ends. The pTY-S VIGS vector plasmid was digested with SnaB I restriction endonuclease. Positive clones were identified using amplification of the TYMV-CP gene of the expected size (522 bp). Ten μL of purified pTY-S carrying the target gene plasmid DNA was injected into the root flesh of red fleshed radish. Plants injected with empty pTY-S vector were used as controls. Injected plants were maintained in an artificial climate chamber at 25°C/22°C with a 16 h/8 h light/dark cycle. The phenotype was assessed after three weeks. Primers used to detect clones that silence the *LINC15957* gene are listed in **Supplementary Table 1**.

### 2.5 Determination of anthocyanin content

The pH difference method was performed as described by Mao et al. (2021). 1 g of the samples were well ground in liquid nitrogen, 0.05% hydrochloric acid monomethanol solution (pre-chilled at 4°C) was added with full tube, mixed well and placed in the dark at 4°C. Two test tubes were set up (1 ml of extract in each tube), and 4 ml of 0.4 mol/L KCl-HCl buffer (pH 1.0) and 0.4 mol/L citric acid/disodium hydrogen phosphate buffer (pH 5.0) were added, mixed well and allowed to stand for 20 min at room temperature. The 510 nm and 700 nm absorbance values were measured by a UV spectrophotometer. Each sample was repeated with three times.

### 2.6 Gene expression analysis

The PCR and amplification was conducted as described previously (Luo et al., 2020). Real-time quantitative PCR analysis (RT-qPCR) was performed on SYBR Green Master Mix (GeneStar, Hangzhou, China) using a BoriPlantGene 9600 PlUS (FOD-96A) Fluorescence Quantitative (Hangzhou, China). The radish *Actin* gene was used as an internal control. The relative expression levels of candidate genes were determined using Equation 2^-ΔΔCt^. Three biological replicates were performed for all reactions. All primers are listed in **Supplementary Table 1**.

### 2.7 Transcriptome sequencing and data analysis

The total RNA was extracted from the LINC15957 overexpressed plants and control radish leaves using RNAprep Pure Plant Kit (TIANGEN, Beijing, China). A cDNA library was constructed for each sample using enriched mRNA. The libraries were sequenced using the Illumina HiSeq-PE150 sequencing platform from BeijingNovogene Co. Ltd. Reference genome and gene model annotation files were downloaded from genome website directly (http://39.100.233.196:82/download_genome/Brassica_Genome_data/Rapsa_Xiang_V1.0/). Index of the reference genome was built using Hisat2 v2.0.5 and paired-end clean reads were aligned to the reference genome using Hisat2 v2.0.5.

Differential expression analysis of two processing samples (three biological replicates per process) was performed using the DESeq2 R package (1.20.0). Gene Ontology (GO) enrichment analysis of differentially expressed genes was implemented by the clusterProfiler R package, in which gene length bias was corrected. GO terms with corrected Pvalue less than 0.05 were considered significantly enriched by differentially expressed genes. ClusterProfiler R package was used to test the statistical enrichment of differential expression genes in Kyoto Encyclopedia of Genes and Genomes (KEGG) pathways.

### 2.8 Statistical analysis

All anthocyanin determinations and real-time quantitative polymerase chain reaction (RT-qPCR) were performed in triplicate, and the data are expressed as the mean ± standard deviation (SD). Student’s t test was used to compare different samples. A difference was considered statistically significant when P < 0.05 or very significant when P < 0.01.

## 3 Results

### 3.1 Acquiring and cloning of *LINC15957*

**Figure 1:**
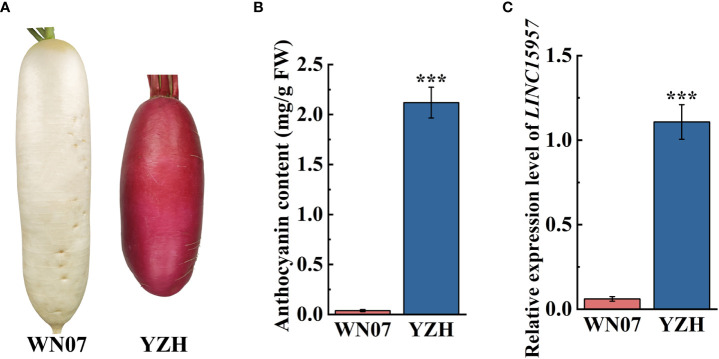
Anthocyanin content and related gene expression in radish with different colors. **(A)** Two radish cultivars “WN07” and “YZH” with different colored radish. **(B)** Content of anthocyanin in root skin of “WN07” and “YZH” radish. **(C)** The relative expression of *LINC15957* in “WN07” and “YZH” radish root skin. Error bars represent the mean ± SE of three biological replicates. ***p < 0.001, Student’s t-test.

The anthocyanin was accumulated in the root skin and flesh stem of “YZH” radish (2.12 mg/g), while almost no anthocyanin was accumulated in white skinned and flesh radish “WN07” (0.04 mg/g) (**Figures 1A, B**). To explore lncRNAs involve in regulation of anthocyanin biosynthesis in radish, “YZH” and “WN07” was used to lncRNA sequencing. The result showed that *LINC15957* were differentially expressed lncRNA in “YZH” and “WN07” (data not shown). RT-qPCR results showed that *LINC15957* was highly expressed in the root bark of red skinned and flesh radish, while the expression level was lower in white skinned and flesh radish (**Figure 1C**). According to genome and transcriptome sequences, primers were designed to amplify the full length of *LINC15957*, a 314-bp fragment were obtained (**Supplementary Table 2**).

### 3.2 *LINC15957* positively regulates the biosynthesis of anthocyanin in radish

**Figure 2:**
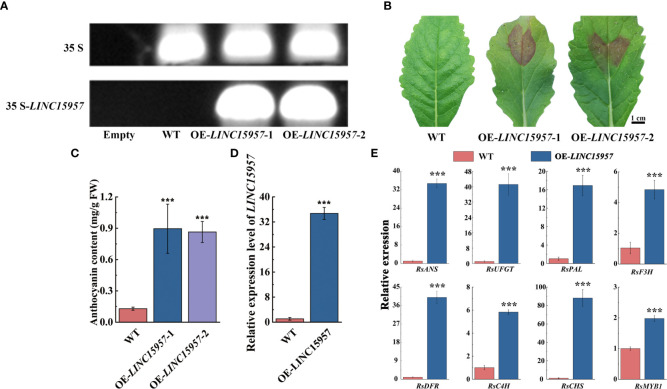
The transiently expressed *LINC15957* in radish leaves. **(A)** The PCR validation of *LINC15957* in OE-*LINC15957* and control. The upper band indicates 35S and the lower band indicates *LINC15957* is successfully transiently expressed in radish leaves. “Empty” is leaves without injection infection, “WT” is empty vector. **(B)** *LINC15957* is transiently expressed in radish leaves. **(C)** Anthocyanin content in the tissue of injection empty, OE-*LINC15957*-1 and OE-*LINC15957*-2. **(D)** The gene expression level in OE-*LINC15957*. **(E)** Expression pattern of key anthocyanin biosynthetic genes in OE-*LINC15957*. *RsACTIN* was used as reference gene for normalization. Error bars represent the mean ± SE of three biological replicates. ***p < 0.001, Student’s t-test.

To further investigate the role of *LINC15957* involve in the anthocyanin biosynthetic pathway, the overexpressed vectors of *LINC15957*-pGreenII62-SK with the constitutive 35S promoter was constructed. Two cotyledon of radish were selected for transient overexpression of *LINC15957* and pGreenII62-SK (control). PCR amplification indicated that *LINC15957* was successfully transferred into radish leaves (**Figure 2A**). The pigmentation was observed in injection sites of radish leaves after transformation with *LINC15957* (**Figure 2B**). The anthocyanin content of OE- *LINC15957* plants was significantly higher than that of the empty vector 6 - 7 times (**Figure 2C**). The gene expression level of *LINC15957* in OE-*LINC15957* plants was almost 32.5-fold greater than in control (**Figure 2D**). The relative expression level of the anthocyanin biosynthetic genes was conducted by qRT-PCR. The results showed that a significant increase in expression of *RsANS* (anthocyanidin synthase), *RsUFGT* (UDP-glucose:flavonoid 3-O-glucosyltransferase), *RsPAL* (phenylalanine aminolyase), *RsF3H* (flavanone 3β-hydroxylase), *RsDFR* (dihydroflavonol 4-reductase), *RsC4H* (Cinnamate 4-Hydroxylase) and *RsCHS* (chalcone synthase), when *LINC15957* were overexpressed. In addition, the expression level of *RsMYB1* in OE-*LINC15957* plants was 2.0-fold greater than in control (**Figure 2E**).

### 3.3 *LINC15957* silencing reduced anthocyanin accumulation in radish root flesh

**Figure 3:**
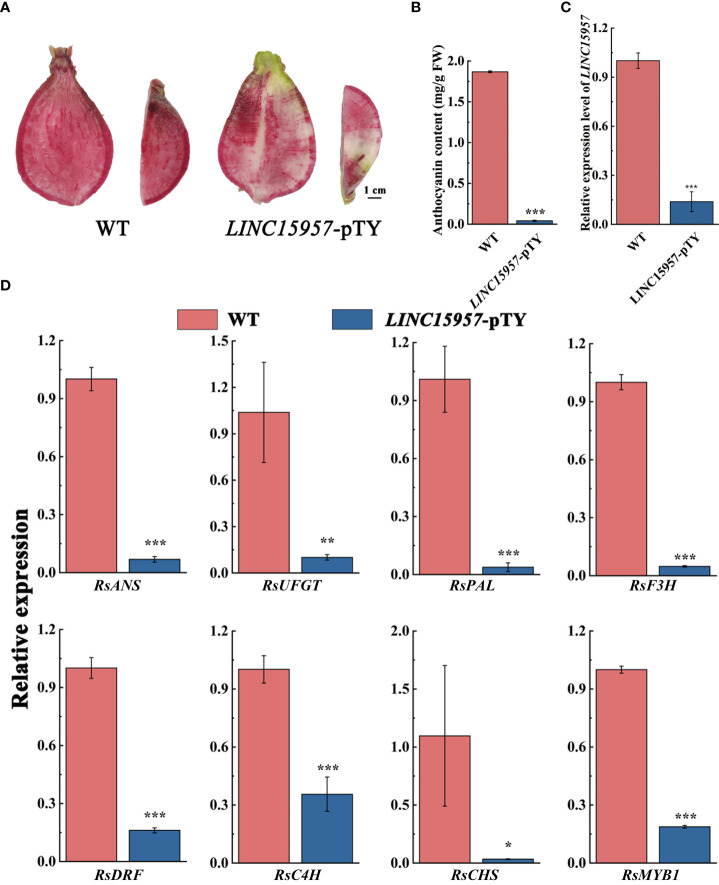
Viral induced silencing of the *LINC15957* genes in radish flesh. **(A)** Radish were inoculated with pTY (WT) or *LINC15957*-pTY (from left to right). Photographs of root were taken at 4 week post-inoculation. **(B)** Anthocyanin content in the tissue of injection empty and *LINC15957*-pTY. **(C)** The gene expression level in *LINC15957*-pTY. **(D)** Expression pattern of key anthocyanin biosynthetic genes in *LINC15957*-pTY. *RsACTIN* was used as reference gene for normalization. Error bars represent the mean ± SE of three biological replicates. *p < 0.05, **p < 0.01, ***p < 0.001, Student’s t-test.

*LINC15957*-pTY recombinant plasmid is used to silence the expression of *LINC1595* in red skin and flesh radish. Decreased coloration was displayed at the infiltration sites 4 weeks after transformation with *LINC15957*-pTY, while no color change was observed with transformation of pTY (**Figure 3A**). The anthocyanin content in *LINC15957*-pTY was significantly lower than that of the control (**Figure 3B**), indicating the accumulation of anthocyanin in the root flesh of radish infected with *LINC15957*-pTY was inhibited. The expression level of *LINC15957* in fruit infected with *LINC15957*-pTY was significantly lower than that of the control (**Figure 3C**). In comparison with the control, the anthocyanin related structural genes (*RsANS*, *RsUFGT*, *RsPAL*, *RsF3H*, *RsDFR*, *RsC4H* and *RsCHS*) in fruit infected with *LINC15957*-pTY was significantly decreased (**Figure 3D**). The expression level of *RsMYB1* in *LINC15957*-pTY were reduced in comparison with the control. Taken together, these results demonstrate that *LINC15957* is positively regulate anthocyanin synthesis in radish.

### 3.4 Transcriptome sequencing and DGE analysis

**Figure 4:**
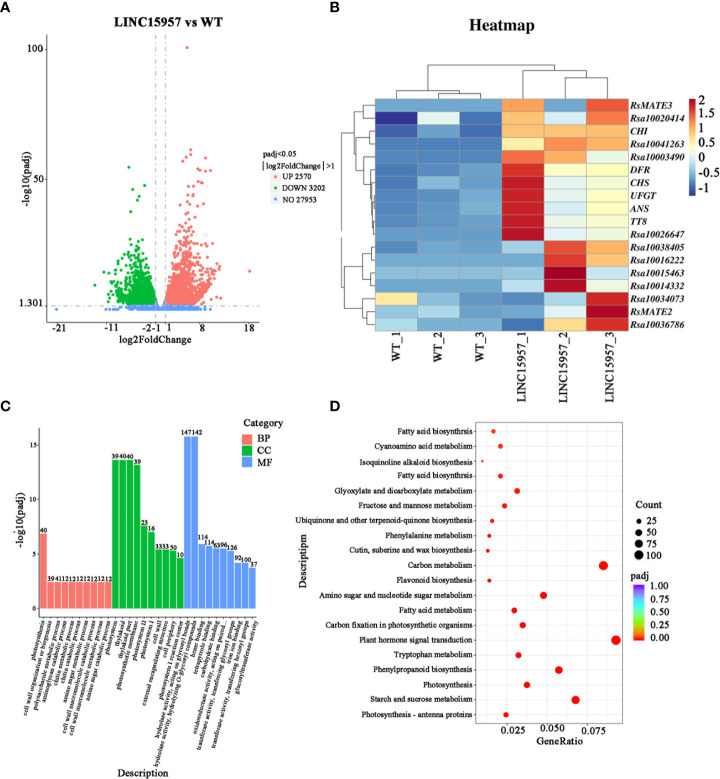
Transcriptome analysis in WT vs. OE-*LINC15957*. **(A)** Volcano plot showing the differentially expressed genes (DEGs) in red and green. The X-axis represents the fold change in WT vs. OE-*LINC15957* (on a log_2_ scale). The Y-axis represents the negative log_10_ transformed average FPKM values. **(B)** Expression levels of the 6 core genes and 12 transcription factors involved in anthocyanin biosynthesis in radish leaves. Each square represents the transcription level in the two processes (WT and overexpressing *LINC15957*) at radish leaves. Different color indicates differences in the level of gene expression, from high (red) to low (blue). **(C)** Gene Ontology (GO) enrichment analysist for differentially expressed genes (DEGs) between WT and OE-*LINC15957*. **(D)** Kyoto Encyclopedia of Genes and Genomes (KEGG) enrichment analysis of the DEGs.

To further identify genes regulated by *LINC15957* in radish anthocyanin accumulation, six libraries (*LINC15957*-overexpressed plants and control) were constructed for transcriptome sequencing. The raw data (Q20 ≥ 97.09% and Q30 ≥ 92.17%) for overexpression and control were 6.68 Gb and 7.06 Gb, respectively. After trimming the barcode, 6.53 and 6.89 Gb of clean data were obtained (**Supplementary Table 3**), respectively. The average for all samples with GC percentage was 46.78% (**Supplementary Table 3**). Approximately 85% of the clean reads were mapped to 38,022,061 genes in radish reference genome (**Supplementary Table 4**), of which 3,547 genes were found to be novel loci that were not annotated in the radish genome (**Supplementary Table 5**). To investigate DEGs and anthocyanin biosynthesis or regulatory genes in the candidate region, we compared the transcriptomes of *LINC15957*-overexpressed plants and WT. A total of 5,772 differential genes were identified, including 2,570 up-regulated and 3,202 down-regulated genes (**Figure 4A**). Early genes include PAL, CHS, F3H, chalcone flavonol ketone isomerase (CHI), flavonoid 3′-hydroxylase (F3′H) and Flavonoid 3’5’-hydroxylase (F3′5′H), which contribute to the formation of dihydroflavonol. The late genes DFR, ANS, UFGT, methyltransferase (MT), and rhamnosyltransferase (RT) plays a role in anthocyanin production. In the present study, we identified 12 core genes that are up-regulated and associated with anthocyanin biosynthesis. Notably, both early (PAL, F3H and CHS) and late (DFR, ANS and UFGT) genes were significantly up-regulated in *LINC15957*-overexpressed plants compared to controls (**Figure 4B**).

To further identify the function of DEGs, Gene Ontology (GO) term and Kyoto Encyclopedia of Genes and Genomes (KEGG) pathway enrichment analyses of the identified DEGs were carried out. GO annotations revealed that all DEGs were assigned to 76 GO terms. In the molecular function (39 terms), the major subcategories were hydrolase activity, heme binding, tetrapyrrole binding. For biological process (21 terms), photosynthesis, cell wall organization or biogenesis and polysaccharide metabolic process were the dominant terms. The ‘photosystem’ and ‘thylakoid’ and ‘thylakoid part’ terms were extraordinarily remarkable in the cellular component (16 terms) (**Figure 4C**). KEGG pathway enrichment analysis showed that tyrosine, phenylalanine, tryptophan, phenylpropanoid, flavonoid biosynthesis was significantly enriched, which indicated that anthocyanin were synthesized from the precursor phenylalanine by the biosynthesis of phenylpropanoid and flavonoid (**Figure 4D**).

### 3.5 Transcription factors involve in anthocyanin biosynthesis

**Figure 5:**
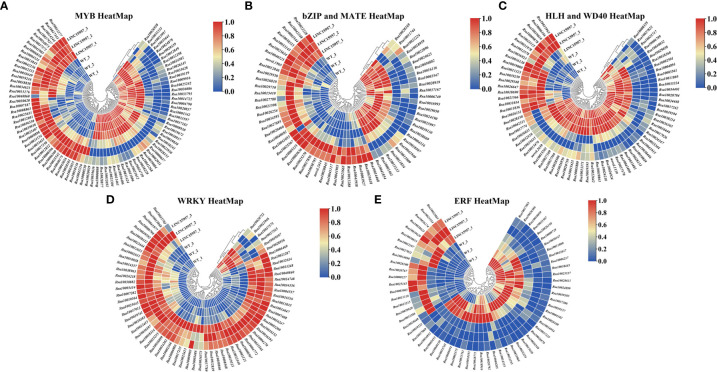
Heat-map depicting normalized log_2_-fold changes in mRNA expression inferred from RNAseq data for transcripts involved in transcription factor family. **(A)** Differential MYB transcription factor heat-map. **(B)** Differential bZIP and MATE transcription factor heat-map. **(C)** Differential HLH and WD40 transcription factor heat-map. **(D)** Differential WRKY transcription factor heat-map. **(E)** Differential ERF transcription factor heat-map.

Many studies have indicated that many transcription factors were involved in anthocyanin biosynthesis, such as MYB, bHLH, WD40 (Liu et al., 2018b), basic domain leucine zipper (bZIP) (Fan et al., 2019), MATE (Gomez et al., 2011), WRKY (Cong et al., 2021) and ethylene responsive factor (ERF) (Wu et al., 2020b). Transcriptome analysis in *LINC15957*-overexpressed revealed 12,167 transcription factors were identified, of which 3,849 were found to be differentially expressed (**Supplementary Tables 6**, **7**), of which 1,934 were upregulated and 1,915 were downregulated. A total of 87 MYB transcription factors were identified, of which 55 were upregulated (**Figure 5A**). Totally, 56 bHLH transcription factors were detected, of which 13 were upregulated. A total of 16 WD40 transcription factors were identified, of which 6 were upregulated (**Figure 5B**). A total of 27 bZIP and 36 MATE transcription factors, of which 6 and 18 were upregulated, respectively (**Figure 5C**). In all, 67 WRKY transcription factors, of which 61 were upregulated (**Figure 5D**). A total of 61 ERF transcription factors, of which 19 were upregulated (**Figure 5E**). Most of the transcription factors involved in the regulation of genes related to the anthocyanin biosynthetic pathway belong to the MYB, bHLH, and WD40 families. These transcription factors identified in this study may be involved in anthocyanin accumulation in radish.

### 3.6 RT-qPCR assay

**Figure 6:**
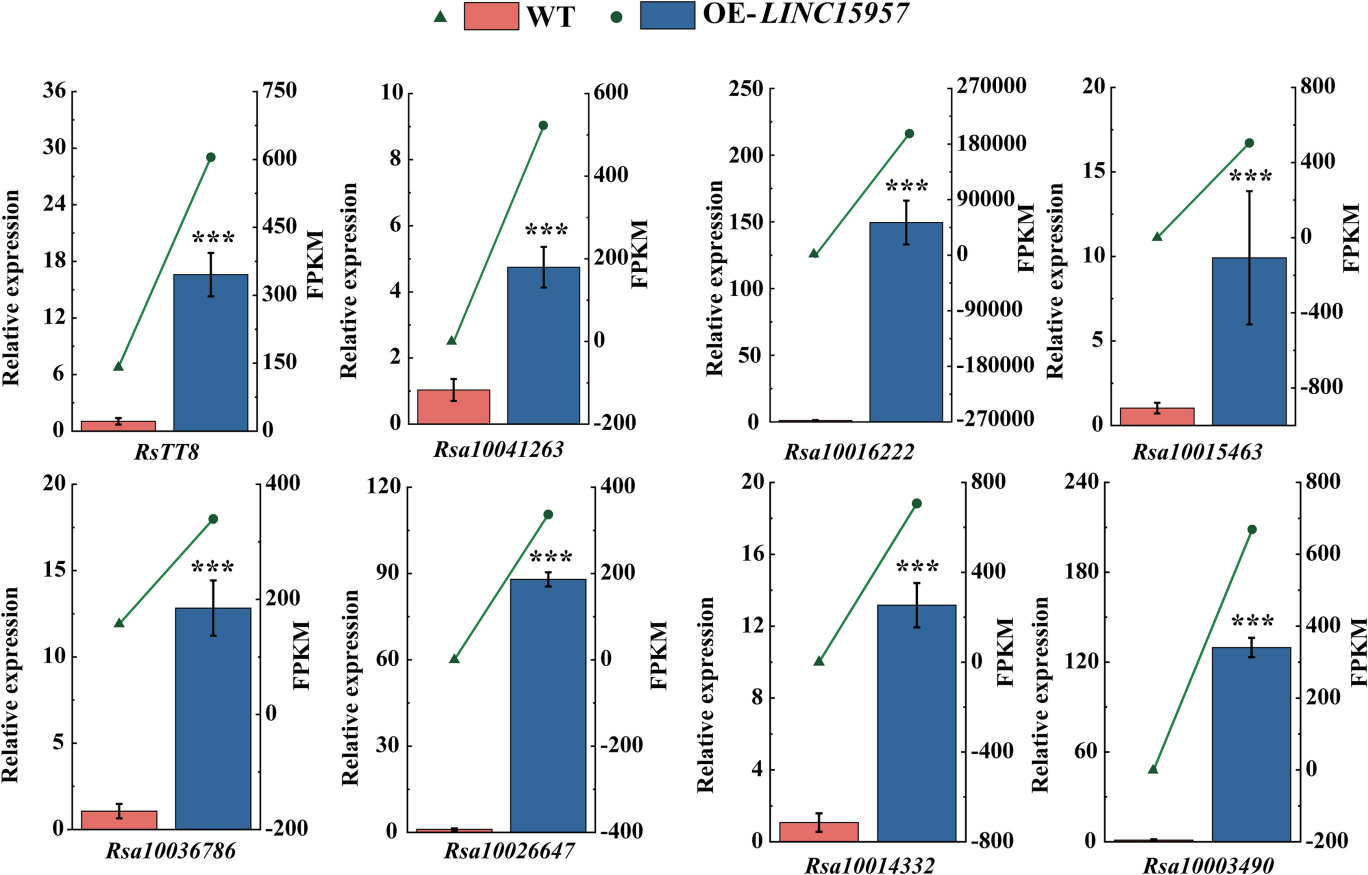
The genes related to anthocyanin biosynthesis in OE-*LINC15957* were analyzed by RT-qPCR. Relative gene expression levels were standardized relative to actin transcription levels. Error bars represent the mean ± SE of three biological replicates. ***p < 0.001, Student’s t-test.

To determine *LINC15957* regulates anthocyanin accumulation in radish, the expression of anthocyanin biosynthetic structure gene and transcription factors in *LINC15957*-overexpressed plants was analyzed by RT-qPCR. A total of 8 genes were selected to validated (**Figure 6**). The expression levels of these 8 genes in different treatments were consistent with those determined by RNA-Seq, indicating the reliability of the transcriptome sequencing results. It was found that the expression level of all 8 genes in *LINC15957*-overexpressed plants was higher than that in control. Therefore, these results indicate that the 8 genes can be regulated by *LINC15957* through the radish anthocyanin biosynthesis pathway.

## 4 Discussion

The anthocyanin-rich radish varieties have good appearance quality, and also have high nutrition and value. The genes related to anthocyanin synthesis in radish have been widely characterized (Lim et al., 2016; Lai et al., 2021). It was found that genes homologous to Arabidopsis *AtPAP1/2* in radish were involved in the regulation of radish anthocyanin synthesis (Lim et al., 2016). *RsMYB1* was shown to be a key transcription factor regulating anthocyanin synthesis by heterologous overexpression in Arabidopsis, tobacco, and Petunia (Lim et al., 2016; Ai et al., 2017). The key gene *RsMYB1.1* controlling the purple skin trait of radish fleshy roots was identified using QTL-seq and QTL mapping, and identified four homologs of Arabidopsis *PAP1* (*RsMYB1.1* to *RsMYB1.4*) in the radish genome (Liu et al., 2019a). *RsMYB1*, a key gene controlling anthocyanin biosynthesis in the fleshy root of radish was mapped by combined QTL-seq and RNA-seq technique, and verified their function by VIGS (Wang et al., 2020b). The key gene *RsMYB90* was identified, which controls the red-skinned trait of fleshy roots of radish (Luo et al., 2020). Transient overexpression of *RsGST1* and the key anthocyanin biosynthesis regulator *RsMYB1a* in radish leaves were significantly enhanced anthocyanin biosynthesis. Dual luciferase and yeast single hybridization assays showed that *RsMYB1a* binds to the promoter and activates *RsGST1* expression. However, the molecular mechanisms regulating radish anthocyanin biosynthesis are not yet resolved.

**Figure 7:**
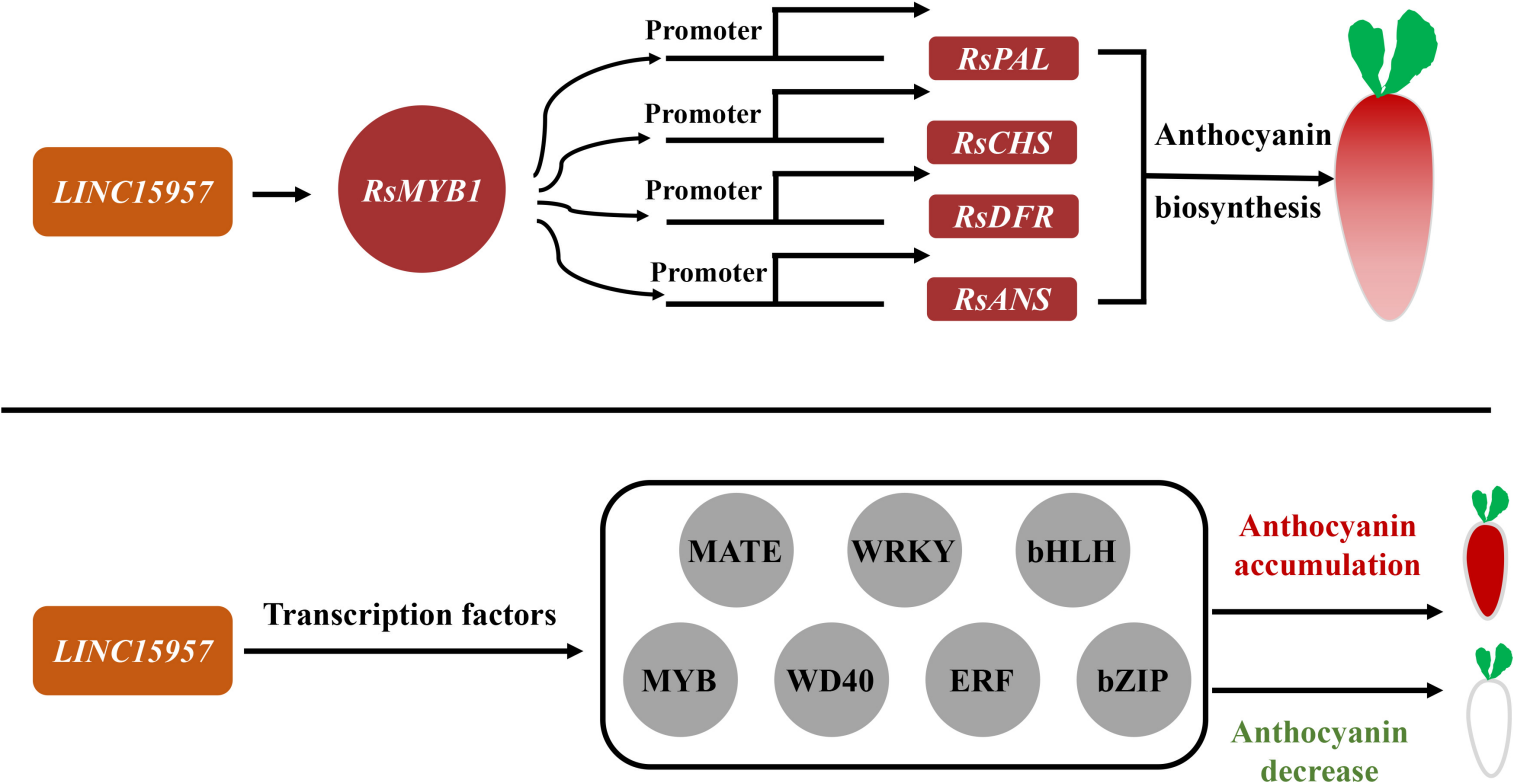
The regulatory pattern of LINC15957 with transcription factor and anthocyanin synthesis-related genes. Red represents up-regulation, gray represents up-regulation or down-regulation, rectangle represents genes related to anthocyanin synthesis, and circle represents transcription factors.

Long noncoding RNAs are a potential regulator in pigment formation (Jin et al., 2013) and can activate multiple mechanisms or affect distal genes in a trans manner (Ørom et al., 2010; Ariel et al., 2014). Many studies found that lncRNAs recruited epigenetic complexes or act as target mimics (Liu et al., 2015; Yang et al., 2019). It was shown that the lncRNA mutant *lncRNA1459* associated with tomato ripening significantly reduced lycopene accumulation and decreased the expression of related genes. It is predicted that *lncRNA1459* indirectly regulates gene transcription by interacting with target proteins (Li et al., 2018). LncRNAs *MLNC3.2* and *MLNC4.6* act as endogenous targeting mimics (eTM) of miR156a and promote anthocyanin accumulation in apple fruit (Yang et al., 2019). The expression of lncRNA-*MdLNC610* was activated under light treatment, which in turn activated the expression of *MdACO1* and increased ethylene production and anthocyanin accumulation in apple fruits. Overexpression of *MdLNC499* promoted anthocyanin accumulation and induced the expression of *MdERF109* and *MdWRKY1* (Ma et al., 2021). *MdLNC610* was physically located downstream with the 1-aminocyclopropane-1-carboxylate oxygenase (ACO) ethylene biosynthesis gene *MdACO1* and affected anthocyanin biosynthesis under high-light treatment (Yu et al., 2022). In this study, the anthocyanin accumulation was increased in transient overexpression of *LINC15957* in radish leaves, while anthocyanin accumulation was significantly reduced in red flesh radish root by silencing *LINC15957*. A total of 5,772 differentially expressed genes were identified in transcriptome data. The anthocyanin related structural genes (*RsANS*, *RsUFGT*, *RsPAL*, *RsF3H*, *RsDFR*, *RsC4H* and *RsCHS*) and *RsMYB1* were changed significantly with anthocyanin (**Figure 7**). KEGG enrichment analysis revealed the genes were significant enriched in tyrosine, L-Phenylalanine, tryptophan, phenylpropanol, and flavonoid biosynthesis. These results suggested that *LINC15957* can play an important role in anthocyanin accumulation.

Anthocyanin biosynthesis in plants was regulated by various transcription factors, such as MYB, bHLH, WRKY, ERF, bZIP and BBX family members (Gonzalez et al., 2008; Jaakola et al., 2010). The transcriptional activator MBW complex (MYB-bHLH-WD40) regulates the activation of genes associated with anthocyanin biosynthesis in plants (Zhao et al., 2013). The R2R3 MYB transcription factor was involved in a variety of plant functions, including activation or repression of anthocyanin biosynthesis (Niu et al., 2010; Zhao et al., 2013; Chen et al., 2022). In general, bHLH transcription factors regulated structural genes responsible for anthocyanin biosynthesis (Zhang et al., 2019; Deng et al., 2020). *CmbHLH2* and *CmMYB6* interaction played a key role in anthocyanin pigmentation changes in *chrysanthemum* leaves and seeds (Lim et al, 2021). Overexpression of *PbbHLH2* in “Red Zaosu” increased anthocyanin content and the gene expression levels of late biosynthetic genes. The WD40 repeat protein in *Medicago truncatula* is required for tissue-specific anthocyanin and proanthocyanidin biosynthesis but not for trichome development (Pang et al., 2009). Light promoted anthocyanin accumulation in dendrobium seedlings through the upregulation of the WD40 repeat transcription factor *DcTTG1*, which induced the expression of anthocyanin synthesis-related genes (Jia et al., 2021). In Arabidopsis, the WD40 repeat protein *TTG1* forms a complex with bHLH (GL3, EGL3, or TT8) and R2R3-MYB (*PAP1*, *PAP2*, *MYB113*, and *MYB114*) TFs regulated anthocyanin synthesis (Zhang et al., 2003; Gonzalez et al., 2008). It was found that *RsMYB41*, *RsMYB117*, and *RsMYB132*, homologs of *AtPAP1/2*, were highly expressed in the root bark of red-skinned radish fleshy roots, while *RsMYB65* and *RsMYB159* were highly expressed in the root bark of purple-skinned radish fleshy roots, and presumably involved in the regulation of the anthocyanin synthesis of radish fleshy roots. The expression analysis revealed that two of the 135 bZIP gene family members (*RsbZIP011* and *RsbZIP102*) might be involved in the regulation of anthocyanin biosynthesis in radish (Fan et al., 2019). Transcriptome analysis revealed that some members of WRKY, ERF, GRAS, NF-YA, C2H2-Dof, HD-ZIP, AP2, zinc finger protein, Tify, HB and LBD gene families may be involved in the regulation of radish anthocyanin biosynthesis (Sun et al., 2018; Gao et al., 2019; Yu et al., 2020; Gao et al., 2020b). In this study, A total of 87 MYB, 56 bHLH,16 WD40, 27 bZIP, 36 MATE, 67 WRKY and 61 ERF transcription factors were differential expression in OE-*LINC15957* plants (**Figure 7**). These results suggested that transcription factors could involve in the regulation of anthocyanin accumulation in radish.

## 5 Conclusion

In this study, the overexpression of *LINC15957* were improved anthocyanin accumulate in radish leaves, and silence of *LINC15957* were decreased anthocyanin accumulate in red-fleshed radish by VIGS, indicating *LINC15957* were positively regulates anthocyanin accumulate in radish. Transcriptome sequencing analysis revealed that the genes *ANS*, *PAL*, *C4H*, *CHS*, and *DFR*, which are related to anthocyanin biosynthesis, were differentially expressed in *LINC15957* overexpressed leaves. RT-qPCR results also verified that these structural genes were highly expressed in *LINC15957* overexpressed leaves, demonstrating that *LINC15957* plays important roles in radish anthocyanin biosynthesis. These results in the present study provide a theoretical basis for understanding the regulatory mechanisms of lncRNAs involve in anthocyanin biosynthesis and breeding varieties with high anthocyanin content in radish.

## Data availability statement

The datasets presented in this study can be found in online repositories. The names of the repository/repositories and accession number(s) can be found below: https://www.ncbi.nlm.nih.gov/, PRJNA916343.

## Author contributions

HT: writing original draft preparation. HT and XL: writing – review and editing. HT, XL, JLu, LW, YL, YJ and XP: methodology. XX, JLi and WZ: resources. HT, XL and JLu: data analysis. All authors contributed to the article and approved the submitted version.
