# Supplementary material for: The long noncoding RNA LINC15957 regulates anthocyanin accumulation in radish
Source: Front Plant Sci. 2023 Feb 27;14:1139143. doi: 10.3389/fpls.2023.1139143 (PMC10009236; doi:10.3389/fpls.2023.1139143)
Supplement: Supplementary file 1 [file Table_1.docx]

**Supplementary Table 1 Sequence of specific primers used in this study**

| **Primer use** | **Primer name** | **Primer sequence (5’–3’)** |
| --- | --- | --- |
| **Gene cloning** | *LINC15957*-F  *LINC15957*-R | CGGGATCCGATACACGCTATGGTATCACAT  GGGGTACCTTGATACGAGCCGACTGCGCTC |
| **RT-qPCR** | *LINC15957*-QF  *LINC15957*-QR | ACACGCTATGGTATCACA  AATAAGGTTAATGCTGGAAGAT |
|  | *RsC4H*-QF  *RsC4H*-QR | AAGGAAGTGAAGGAGAAGAG  CGTTAGTTGTGGCTTGTG |
|  | *RsCHS*-QF  *RsCHS*-QR | CATAGATGGACACTTGAG  CAGTCGCTTATACCTAAC |
|  | *RsUFGT*-QF  *RsUFGT*-QR | CAAGTTCTGCGATTACAT  AAGGCTTATCAAGTTCATC |
|  | *RsDFR*-QF  *RsDFR*-QR | CGATTCTTAGCATCTCAG  ATTCCACCAACATATCCT |
|  | *RsANS*-QF  *RsANS*-QR | CTTCATTCTACACAACAT  TAACCTTCTCCTTATTCA |
|  | *RsF3H*-QF  *RsF3H*-QR | TCCTGAGGAGAAACTGAAGTTCG  CGTCACGATCTCTCTCCAATCTT |
|  | *RsMYB90*-QF  *RsMYB90*-QR | GCAGGAAGAGTTGTAGAC  ATGAAGGCGAAGAAGAAG |
|  | *RsPAL*-QF  *RsPAL*-QR | CTCCATAATCTCCTCCAT  CATCGTGACATTCTTGAT |
|  | *Rsa10041263*-QF  *Rsa10041263*-QR | TTGTGGAAGATTGCTACT  GCCAATGATGAAGAAGAG |
|  | *Rsa10026647*-QF  *Rsa10026647*-QR | GTGCGACATCTACTGATA  CACCTTCTTCCTCTTCTC |
|  | *Rsa10003490*-QF  *Rsa10003490*-QR | GCTACTTGGCTTCTGTAA  AACGCTTCTCTTCATCAA |
|  | *Rsa10015463*-QF  *Rsa10015463*-QR | CAACAACAAGGTGACAAT  GGAGTAGAAGAGGAACTG |
|  | *Rsa10016222*-QF  *Rsa10016222*-QR | ATACAGCCAATCTTCCAA  GACCACATAGTCCTTCTT |
|  | *Rsa10036786*-QF  *Rsa10036786*-QR | TATCATCAGATTAGAAGGAGAG  AACAACGACGAAGAAGAT |
|  | *Rsa10014332*-QF  *Rsa10014332*-QR | GAAGCAATAACCGAACTT  AATACTCATTCATAGCCTTG |
| **Transient overexpression** | *LINC15957*-TF  *LINC15957*-TR | CGGGATCCGATACACGCTATGGTATCACAT  GGGGTACCTTGATACGAGCCGACTGCGCTC |
| **Transient overexpression verification** | 35S-F  35S-R | CATTGCGATAAAGGAAAGGC  CGAAGGATAGTGGGATTGTGC |
|  | PBI121(35S)  LINC-R | GACGCACAATCCCACTATCC  TTGATACGAGCCGACTGCGCTC |
| **gene silencing** |  | ATGATTTGTGTAACCCTATATAAAGAGATCTAATTATCAATTGATAATTAGATCTCTTTATATAGGGTTACACAAATCAT |
| **gene silencing verification** | CP-F  CP-R | TCCACCCTCACCACCTTCTACCG  GTGTGGGGACAGACCTCGCTAACT |
