# Supplementary material for: The long noncoding RNA LINC15957 regulates anthocyanin accumulation in radish
Source: Front Plant Sci. 2023 Feb 27;14:1139143. doi: 10.3389/fpls.2023.1139143 (PMC10009236; doi:10.3389/fpls.2023.1139143)
Supplement: Supplementary file 2 [file Table_2.docx]

**Supplementary Table 2 Sequence of *LINC15957***

>*LINC15957*

GATACACGCTATGGTATCACATACGAAAGAAAGAAGATTCAAAAGTAATATTTCCTTAAGCATTAAGAACAGAGTTTTGGTGTTTTTCCTTTTAAGATACTTTAAGTAGTTTTCCTATTATCCGTAGGGTTATGATTTGTGTAACCCTATATAAAGAGATCTAATTATCAATAAGAACTATCTTCCAGCATTAACCTTATTCTCAAGAGAGACTAGGGTTAAAGGAAGCTTTGCAATCAAGTTATCTCTGTTCTTCTTTGTTCTTACCTAATCACGGCACCACCAGTTACACGAGCGCAGTCGGCTCGTATCAA
